# Fraud within the Nigerian health system, a double threat for resilience of a health system and the response to the COVID-19 pandemic: a review

**DOI:** 10.11604/pamj.2023.45.116.36979

**Published:** 2023-07-06

**Authors:** Monica Ewomazino Akokuwebe, Erhabor Sunday Idemudia

**Affiliations:** 1 Faculty of Humanities, North West University, Mafikeng, South Africa

**Keywords:** COVID-19, fraud, impact, Nigeria, pandemic

## Abstract

As Nigeria battles the COVID-19 pandemic, systemic fraud within the health system may undermine the efforts to halt the devastating effect of the disease and the fight against COVID-19. Fraud is a major concern worldwide, especially in developing countries such as Nigeria, where it is widespread within the health system. The vulnerability of the Nigerian health system despite several efforts from relevant stakeholders, has consistently been underscored before the pandemic arose, raising serious concerns. These concerns include fraud, embezzlement, and mismanagement of funds, exploitation, lack of transparency in policymaking, cutting corners in procurement processes, and taking advantage of the healthcare workforce for personal benefits. Also, other involvements in the vulnerability of the Nigerian health system that are worrisome include stakeholders using the pandemic to their advantage to increase their private benefits, a short supply of vital health resources, fraudulent recruitment of the health workforce, and ineffective crisis management. This study explores fraud within the Nigerian health system, its impact and implications for health-system resilience as well as its response to the COVID-19 pandemic. Guided by agency theory, causes and impacts of fraud in the health system and its implications on the response to COVID-19 were explained. Systematic review method was employed; out of 1462 articles identified and screened dated from 1991 to 2021, sixty articles were included in the analysis and interpretation. Specific fraud interventions should focus on a weak and vulnerable health system, service delivery, high-risk institutionalized health workforce, and addressing issues of fraud within and outside the health system in order to curb the dreaded COVID-19 and its variants in Nigeria.

## Introduction

Despite the growing global anxiety about, and efforts at protecting citizens from the COVID-19 pandemic and the high incidence of mortality from the virus, various studies have revealed that health sectors have encountered numerous challenges with respect to fraud prior to the pandemic. Globally, constructing consensus on drivers of the health sector has been advocated as an action to improve accountability and transparency, which are essential for the growth and capacity of a health system [1-4]. Thus, fraud in the health sector is a key challenge globally, and Nigeria, as part of the worldwide system, is not exempt from systemic fraud in the health sector. Systemic fraud in the health sector refers to the vulnerability of health system to fraud through intentional deception or false representation of health material facts that are committed either for personal gains or for the benefit of some others partly. Therefore, in the war against COVID-19, health system resilience, accountability and integrity are key. Numerous medical staff and specialists in Nigeria have come to the understanding that institutional fraud is a major barrier to reforms in the health system. Thus, fraud is accountable for the lack of progress in the health sector over the past decades [5-7]. Hence, shareholders in the health sector need to be conscious of institutional fraud as the *“deliberate attempt of some individuals to make an institution ineffective by working at cross purposes to its goals, thereby eroding public trust in the institution”*[8-10]. The vulnerability of the Nigeria´s health system to fraud has raised serious apprehensions before the pandemic outbreak but was undermined. Before the COVID-19 pandemic, Nigeria's health system was prone to fraud as a result, patients looked for providers they knew socially and due to financial constraints like unpaid salaries, a lack of opportunities for credible wage hikes, and the slow or stagnant promotion of health workers. All of these issues in tandem motivated a culture of fraud and bribes from which illicit payments became widely accepted as the norm. However, the pandemic was a huge wake-up call for interventions for dilapidated healthcare system in order to curtail the outbreak in Nigeria. However, the pandemic was a huge wake-up call for interventions for dilapidated healthcare system in order to curtail the outbreak in Nigeria [5-10]. Furthermore, health workers are faced with several challenges including only a few test kits and lack of laboratory test equipment [8,9].

The Nigeria Centre for Disease Control Microsite (NCDC) has reported about 70% of infections are occurring in communities without an identifiable trace [10]. The delay in payments of allowances of COVID-19 frontline health workers has made it impossible to join the fight against the virus, and the health and safety of health workers are not being prioritized. Early payment of health workers´ wages is one of the key factors in addressing health emergencies such as the COVID-19 pandemic [11,12]. This can be achieved if there is political will and commitment from government and other relevant health stakeholders. Thus, owing to systemic fraud issues within the Nigerian health system, alongside with the impact of the pandemic, millions of vulnerable Nigerians do not have access to quality healthcare, as 77% of healthcare spending in Nigeria is out-of-pocket. Most Nigerians do not have health insurance of any kind, and the poorest Nigerians have extremely limited access to quality health care [13]. With evident deterioration in the health system over the years, there is not much improvement, particularly in relation to enactment of health policies and financing in the Nigerian health system. Before the COVID-19 pandemic, the government's overall health spending was anticipated to account for 3.6% of the total budget (GDP) in 2015, 3.7% in 2016, 3.8% in 2017, 3.1% in 2018, and 3.0% in 2019 [14,15]. In 2020, government expenditure on the COVID-19 pandemic was 3.38%; in 2021, it rises to 7.0%, with nearly all of the funds sourced from the COVID-19 emergency finance package approved by the International Monetary Fund (IMF). Yet, a pledge of 15% of annual budgets on public health financing has not been achieved over the years [14-17]. This insufficient health budget from the Nigerian government has affected rapid and quick response to the COVID-19 pandemic, and COVID-19 equipment was disproportionately spread across major cities in those states with increased prevalence of COVID-19 victims [16]. The 2020 reports on the COVID-19 management revealed that there were only 169 ventilators, serving an estimate of 1,266,440 persons per ventilator, across the entire country [16,17]. In Nigeria, about 70% of severely dilapidated health centres and outdated wards, shortage of medical essentials and unaffordable drugs, led to outbreaks of cholera and Lassa fever gaining impetus along with COVID-19 sub-variants [15,18]. As Nigeria battles the COVID-19 pandemic, systemic fraud in the health sector may undermine the efforts to combat the devastating effects of the virus [15], as this has been overwhelmed by the rising mortality of persons infected with the virus and its newly emerging variants, as well as other chronic infectious and non-infectious diseases [5,6].

Health fraud, however, affects service delivery considering it may threaten a person's right to equal access to services, contributing to underlying imbalances that limit public access to healthcare services and adversely impact the most vulnerable individuals [2,5]. In addition, widespread healthcare fraud may trigger breakdowns in service delivery chain with devastating implications, including shortage of essential medical supplies. Health stakeholders' inability to perform or unwillingness to stop health fraud might be perceived as a government's inability to effectively deliver medical treatment for its population or deny those residents the opportunity to utilise public services [12,15]. Given their inability to pay for healthcare services, those with lower socioeconomic positions widen the social gap and increase inequality. Consequently, efforts to tackle health fraud in service delivery should be considered an essential aspect of public health campaigns designed to increase the public's understanding of Nigeria's health promotion policies [7,10]. Several studies have shown that health fraud in service delivery has a detrimental impact on human development indicators and accelerates high mortality rates. The Nigerian health system is characterized by a virulent combination of problems including inaccessibility of quality health care, poor hygiene, fraud, malnutrition, lack of access to safe drinking water, poor health infrastructure, fake drugs, insufficient financial investment, and lack of sufficient health personnel [19,20]. The major factors responsible for the poor development of health services in Nigeria include political instability, limited institutional capacity, an unstable economy, corruption, and fraud. Presently, most of the primary health care (PHC) facilities in Nigeria lack the capacity to provide the essential health-care services, in addition to having issues such as inadequate equipment, poor quality of health-care services, poor staffing, poor condition of infrastructure, poor distribution of health workers, and lack of essential drug supply [21,22]. In part, problems with the implementation of PHC in Nigeria are related to the hand-over in the 1980s to the local government administration, which is the weakest level of government [20,23], as the impact of local government administration on the people in Nigeria still remains a subject of intense debate and argument [24]. Hence, actions taken should ensure every Nigerian has access to good and quality healthcare services across the three tiers of healthcare delivery. This study explores the assessment of fraud in healthcare service delivery, its impact and its implications for health system resilience, as well as the response to the COVID-19 pandemic in Nigeria.

## Methods

**Figure 1:**
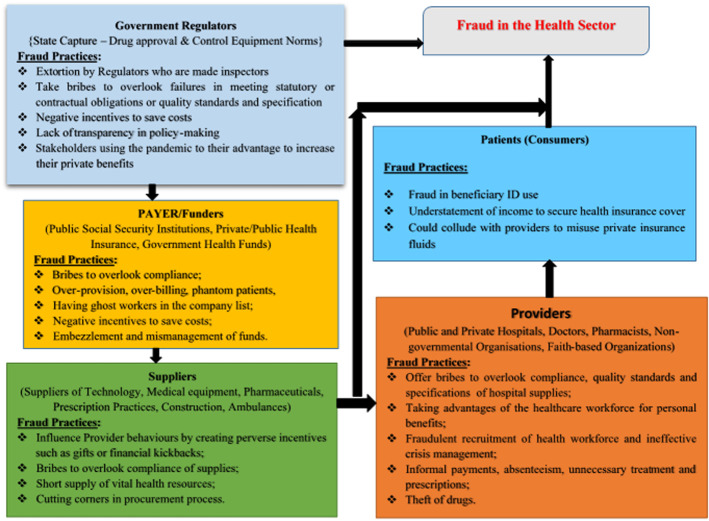
conceptual framework showing the route and actors of fraud in the health sector

**Theoretical/conceptual framework:** this study is anchored on Agency theory propounded by Jensen and Meckling [25], which is used to explain relationships between agents and principals. In their study, the agent represents the principal in a particular business transaction and is expected to represent the best interests of the principal without regard for self-interest, and this has led to the principal-agent problem. In this study, an organizational view was taken where the health sector was simplified into five main actors: government regulators, payer (funders), suppliers, providers, and patients (consumers) [26,27]. From this, we hypothesize how various stakeholders and the nature of their relationships may create opportunities for consumption. The conceptual framework showed classical fraud risks to the supply chain from relationships between these main actors (Figure 1) [28-30]. The possibilities of policy, regulatory, and institutional capture, and of fraudulent interactions between payers and providers tend to be under-researched, even though they have a great impact on the overall performance of the health system [8,30,31]. The flow of funds is illustrated as well as a priori principal-agent relationships that could result in fraudulent practices. Information irregularities in the characteristics of the healthcare system create opportunities for fraud and in the process, healthcare providers may bill patients for services not rendered. Suppliers of medical equipment, technology, or pharmaceutical materials may influence provider behaviour by creating perverse incentives such as gifts or financial kickbacks [3,29,30]. Payers, suppliers, and providers may offer bribes to regulators to overlook failures in meeting statutory obligations or quality standards and specifications. Also, consumers could connive with providers to misuse private insurance funds. In Nigeria, fraud in the areas of distribution and misappropriation of drugs/medical supplies is owing to an under-financed and badly managed system, poor record-keeping, and ineffective monitoring and accounting mechanisms [32-34].

**Figure 2:**
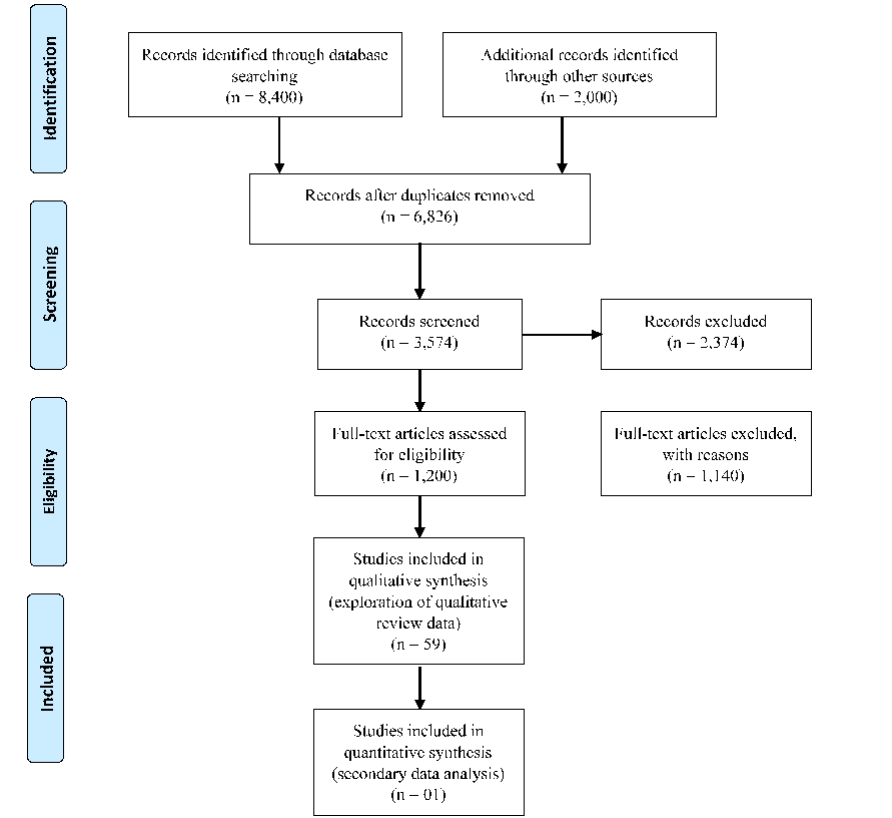
flow diagram showing the selection and sample sizes generated from the selection process

The review adhered to the Preferred Reporting Items for Systematic Reviews and Meta-analyses (PRISMA) statement (Figure 2) [35,36]. Articles included were observational studies, either cross-sectional studies, cohort studies, or case-control, with a publication period between the years 1991 to 2022. The main subject discussed in the articles was fraud, as a double threat for a sustainable health-system resilience and response to the COVID-19 pandemic within the Nigerian health system. Articles which were excluded were those of non-Nigerian origin, or with irrelevant topics being studied, and articles which were not written in English. Official reports from government agencies were not selected as the review included articles with both information on fraud as a double threat for a sustainable health-system resilience and the response to the COVID-19 pandemic within the Nigerian health system. We searched eleven electronic databases which included Scopus, Medline, PubMed, Sage, Google scholar, Embase, Global health expenditure database (GHED), Global health observatory data repository-WHO (GHODR-WHO), Cochrane library, Cumulative index to nursing and allied health literature (CINAHL) and EBSCO-HOST to retrieve studies of potential interest, with inception from 1991 to 2021. The literature search was undertaken using a combination of keywords of health financing OR Nigerian healthcare system, AND fraud within the Nigerian health system, OR health-system resilience AND fraud as a threat to a sustainable health system in Nigeria in the abstract, title, or keywords fields. The search was done in two classifications using the keywords: first, a systematic review of related articles on fraud on Nigerian healthcare system from inception to 1991 to 2022, and second, a review of related articles on the response to the COVID-19 pandemic outbreak amidst fraud within the Nigerian health system gathered from 2020 to 2022. The titles of the study were screened, and the abstracts were analyzed to determine their relevance.

**Study selection:** two researchers were independently recruited to screen titles and abstracts based on inclusion and exclusion criteria. If the inclusion or exclusion criteria could not be decided based on the title and abstract, full-text articles were retrieved, and the decision was made accordingly. Criteria of studies that were sought were study design, study location, sample size, objectives of the study, and major findings, such as fraud within the health system, the implications of fraud in the health system, and the impact of fraud on the response to the COVID-19 pandemic, were extracted and explored. Articles which used community-based studies, cross-sectional studies and secondary data which reported fraud in the Nigerian health system and response to COVID-19 pandemic were included.

**Data extraction:** after the screening of articles, duplicated publications were determined and excluded by comparing authors´ names, study names, and sample size. Study design, sample size and the results of each study were noted. Results selected to be included in the review had to possess the specific assessment of the significant factors in relation to fraud within the health system and how relevant stakeholders could respond to COVID-19. Upon completion of screening and selection of the retrieved studies, a methodological assessment of each study was conducted and followed by extraction of data. The data from the selected studies were extracted according to; i) study overview or characteristics of the study; ii) proportion of the population having specific characteristics on fraud reports on response to the COVID-19 pandemic in Nigeria in a given time period and iii) assessment of fraud in the health system amidst the ongoing COVID-19 pandemic.

**Risk of bias assessment:** the PRISMA critical appraisal tool (PCAT) was used to assess the methodological quality of each observational study. The PCAT was also used to appraise the systematic reviews [37-39]. The PCAT examined the study based on eight criteria which were preliminaries, introduction, design, sampling, data collection, ethical matters, findings/discussion and conclusion. The total score was then converted into percentages and the following categories were assigned to allow for comparison; poor quality (≤ 49%), acceptable quality (50% -74%), and high quality (≤ 75%) [40]. The reason for the comparison categories was to aid in the improving of the reviews reporting and were useful for the critical appraisal of the systematically selected published articles. The preferred reporting items for systematic reviews and meta-analysis (PRISMA) is a 27-item checklist used to improve transparency in systematic reviews. These items cover all aspects of the manuscript, including title, abstract, introduction, methods, results, discussion, and finding.

**PRISMA statement in research reporting:** the reporting of this systematic review was guided by the standards of the preferred reporting items for systematic review and meta-analysis (PRISMA) statement.

## Results

**Study selection:** a total of 10,400 records were identified through searching eleven databases. A total of 3,514 records remained after duplicates were removed. Of those remaining, 1,200 records were deemed ineligible based on their titles and abstracts. Of the 60 papers that were qualified for a full-text review 1,140 full-text articles were excluded as a result that they did not meet the eligibility criteria for the review Figure 2.

**General characteristics of the included study:** sixty peer-reviewed articles were used for the analysis and interpretation of the data. All the selected articles included the assessment of fraud in healthcare service delivery amidst the COVID-19 pandemic and its impact/implications of fraud on health-system resilience and the response to the COVID-19 pandemic in Nigeria as their main findings. General characteristics of the sixty included articles [41-99] and their study findings were summarized in Table 1. Out of the sixty articles included in this study (n= 60), only fifteen articles utilized cross-sectional designs (n= 15), seven articles are of secondary data sources (n= 7) and eight articles were of mixed methods (n= 8). Also, twenty-eight articles employed qualitative/narrative/ systematic/literature review methods (n= 28), fourteen articles were media sources (n= 14) and three articles were commentary sources (n= 3). Fifty-eight articles were conducted in Nigeria (n= 58), while the countries of the remaining two articles (n= 2) could not be ascertained in this study. A majority of these articles (n= 54) were conducted within health settings and eighteen articles (n= 18) were grouped together under the response to COVID-19 and its present situation of the Nigerian healthcare system during the pandemic. Two articles (n= 2) were documented in relation to using COVID-19 leveraging tools to fund Nigeria´s epidemic preparedness towards the pandemic (Table 1).

**Table 1:** summary of the reviewed studies (1991-2022)

Study design	Location	Sample size	Objective	Main findings
Mixed methods	Nigeria	Interviews	investigate purchase and distribution of drugs	impacts of corruption on the health sector are numerous
Qualitative	Nigeria	Self-referred users	factor, perceptions, and experiences of service-users	predisposing factors, Enabling factors and need factors
Narrative review	Nigeria	Systematic review	examine the effects of corruption in Nigeria	corruption and political inequality corruption was found
Descriptive	Nigeria	Civil servants	patterns of self-referral practices among civil servants	civil servants practiced self-referral under the NHIS
Literature review	Nigeria	Intervention	role of ethnicity and religious affiliation	target president's ethnic group, zone, or state of origin.
Descriptive	Nigeria	305,000 individuals	examine the financial burden of OOP health payments	16.4% of households incurred catastrophic health payments
Literature review	Nigeria	Review	public financing for UHC	reduces financial impoverishment
Systematic review	Nigeria	Publications	develop and implement health financing strategies	UHC will catalyse a robust health-care market
Literature review	Nigeria	Publications	Develop health financing and Covid-19	A robust health-care facilities and response to Covid-19
Systematic review	Nigeria	Publications	systematically analyse relevant quantitative studies	health care in Nigeria is financed through different sources
Review	Nigeria	Publications	examines government finance, budgetary allocation	Nigeria also lags behind relative to some other countries
Media sources	Nigeria	Media	examine the presidential summit on UHC in Abuja	political commitment towards health and garnering.
Secondary data	Nigeria	Media	evaluation of COVID-19 outbreak updates in Nigeria	confirmed 263,322 cases, 257253 discharged & 3,148 deaths
Secondary data	Nigeria	Media	effect of COVID-19 on medical education in Nigeria.	the disruption of educational services due to lockdown
Secondary data	Nigeria	electronic databases	the preparedness of Nigeria for COVID-19 pandemic	the Nigerian healthcare system is dilapidated and weak
Media sources	Nigeria	Media	examine the Nigerian budget of ₦2000	prioritize health & investing in Human Capital Development
Media sources	Nigeria	Media	examine the cutting of healthcare spending by 40%	Nigeria's budget for health is unacceptably low, under 5%
Review	Nigeria	electronic databases	examine the funding of the national health-care	Nigeria's longstanding poor health care financing
Commentary	Nigeria	Commentary	examine COVID-19 and progress towards UHC	this situation weighs most heavily on the poor
Systematic review	Africa	Electronic databases	highlight the impact of COVID-19 pandemic	the fragile health system is overburdened with the pandemic
Media sources	Nigeria	Media	what was known about Nigeria’s 2021 budget	**the 2021 budget is higher by 26% from last year’s budget**
Media sources	Nigeria	Media	examine the Disease Prevention Budget	Nigeria, spent eight naira on same disease prevention
Systematic review	Nigeria	Electronic databases	examine how tertiary hospitals can strengthen PHC	many potential patients are likely to bypass the PHC level
Systematic review	Nigeria	Electronic databases	demonstrate a viable path to UHC	manage their own insurance schemes for UCH coverage
Systematic review	Nigeria	Electronic databases	improved access, quality and efficiency in health care	the implementation of strong actions to tackle corruption
Review	Nigeria	Scientific databases	review the state of the Nigerian health care system	medical intelligence and surveillance represent is needed
Review	Nigeria	Scientific databases	strengthening of Nigerian health system	decentralization and fragmentation of the health system
Commentary	Nigeria	Commentary	HIV funding land-scape	COVID-19, the most devastating pandemic
Review	Nigeria	Scientific databases	to assesses the implementation of the PHC	integrated PHC systems for effective provision of health care
Review	Nigeria	Literature reviews	evolution of health care systems in Nigeria	health manpower training and development, and health care
Review	Nigeria	Scientific databases	reposition future health policy in Nigeria for UHC	address historical intractable challenges with new narrative
Media sources	‒	Media	corruption perceptions index 2020 in the pandemic	controlling corruption is essential to ensuring a fair response
Reviews	‒	Recent literature.	identify channels for COVID-19 vaccines distribution	unequal distribution, theft, and black markets
Narrative review	Nigeria	Literature review	identify and prioritise feasible responses to corruption	absenteeism, under-the-counter payments
Descriptive	Nigeria	201 respondents	to describe quality of health, insurance subscription	perception of individuals’ state of health and satisfaction
Descriptive	Nigeria	Secondary data	to strengthen the national health system	encompass public and private providers of health services
Narrative review	Nigeria	Literature review	factors glossed over successive government regimes	ensure equitability and accessibility to healthcare services
Media sources	Nigeria	Media	Nigeria health financing system assessment	health financing system assessment was envisioned
Narrative review	Nigeria	Secondary data	problems hindering successful implementation	efficacy of national development plans would be enhanced
Narrative review	Nigeria	Secondary data	problem of medical tourism and development	brain drain syndrome, underfunding, dilapidated structures
Media sources	Nigeria	Media	Nigerian healthcare challenges and recommendations	devise health care reforms
Media sources	Nigeria	Media	to explore collective action on corruption	trial and monitoring impact of policy interventions
Cross-sectional	Nigeria	Quantitative	corruption in the public sector	significant factors stimulating corruption in Nigeria
Narrative review	Nigeria	Literature review	rule of law facing weak anti-corruption institutions	the need to strengthen civil society groups in Nigeria
Descriptive	Nigeria	Quantitative	pattern and reasons for work absenteeism	absence was highest & lowest among nurses and pharmacists
Media sources	Nigeria	Media	facts about healthcare in Nigeria	the country needs to address healthcare
Secondary data	Nigeria	Media	how Nigeria could avoid waste and corruption	governance reforms for predatory public funds
Secondary data	Nigeria	Demographic and health surveys	redress the serious deterioration in healthcare delivery	decreasing gender barriers in national health development
Commentary	Nigeria	Commentary	necessity for a reform of health systems in Nigeria	revision of National constitution & a separate Health Bill
Primary data	Nigeria	Field research	2008 Nigeria Health System Assessment	Nigerian health sector is broad
Media sources	Nigeria	Media	strengthen Nigeria’s health care systems	one of the world’s most underfunded and least robust.
Media sources	Nigeria	Media	systematically identify different types of corruption	solutions to health sector corruption problems identified
Narrative review	Nigeria	Literature review	impediments of corruption on care delivery efficiency	the malicious effects of corruption, and the need to take action
Narrative review	Nigeria	Literature review	effects of corruption on health care processes	high level of corruption in health care processes
Narrative review	Nigeria	Literature review	the institutional corruption and health-sector reforms	institutional corruption places a huge burden on poor people
Descriptive	Nigeria	474 respondents	overall absenteeism rate and its factors	absenteeism highest (domestic staff) and lowest (doctors)
Mixed methods	Nigeria	Survey	transparency and potential vulnerability to corruption	pharmaceutical system marginally vulnerable to corruption
Review	Nigeria	Literature review	sustainable healthcare system in Nigeria	the way forward with challenges of Nigerian health system
Media sources	Nigeria	Media	COVID-19 business booms among inbound travellers	illegal for anyone to collect any money at the airport
Media sources	Nigeria	Media	Nigerian airport workers take bribes from travellers	notable progress is still being made in the area of vaccines.

**Quality assessment:** quality assessment for each study was conducted using the Crowe critical analysis tool; an established and validated tool used in assessing the quality of observational studies (Table 2) [40]. Twenty-three studies were rated to be of high quality [41,43,48-50,53,54,56,58,63, 72,76,78-81,84,85,88,92,94 96, 98] (Table 3), while the remaining thirty-seven studies were of acceptable quality [42,44-47,51,52,55,57,59-62,71,73-75,77,82,83,86,87,89-91,93,95,97,99] (Table 3).

**Table 2:** quality assessment of studies using Crowe critical analysis tool (CCAT)

Author/year	Preliminaries (/5)	Introduction (/5)	Design (/5)	Sampling (/5)	Data collection (/5)	Ethical matters (/5)	Results (/5)	Discussion (/5)	Total (/40)	%
Akokuwebe ME, *et al*. (2017)	4	4	4	4	3	3	4	4	30	75.0
Koce F *et al*. (2019)	2	2	3	3	3	2	3	2	20	50.0
Adegboyega K *et al*. (2012)	3	3	4	3	4	3	4	4	28	70.0
Okoli H *et al*. (2017)	2	2	2	2	3	2	3	3	19	47.5
Chukwuma A, (2019)	2	2	3	2	3	2	3	3	20	50.0
Aregbeshola BS (2018)	2	2	2	2	2	2	2	2	16	40.0
Awosusi A, (2015)	2	2	2	3	3	3	3	3	21	52.5
Uzochukwu BS (2015)	4	3	4	3	3	2	4	4	27	67.5
Akunne MO (2019)	2	2	3	3	4	2	4	4	24	60.0
Adebisi YA (2020)	3	3	3	2	3	1	4	4	27	67.5
Universal health coverage (2020)	2	2	2	2	2	2	2	2	16	40.0
Nigeria centre for disease control (2020)	2	2	2	3	3	3	4	4	23	57.5
Usman F (2021)	3	3	3	3	3	2	4	4	25	62.5
Anyanwu MU (2020)	3	2	3	3	4	3	4	4	26	65.0
Olufemi J( 2020)	2	2	2	2	2	2	2	2	16	40.0
Akinwotu E (2020)	4	3	3	3	4	2	3	3	25	62.5
Aregbeshola BS (2022)	2	2	2	2	2	2	2	2	16	40.0
Amos *et al*. (2020)	3	3	3	3	3	1	4	4	24	60.0
Ogunkola IO (2021)	2	2	3	3	3	2	3	3	21	52.5
Obokoh A (2020)	2	2	2	2	2	2	2	2	16	40.0
Joel-Osoba G (2021)	2	2	2	2	2	2	2	2	16	40.0
This day, (2021)	2	2	2	2	2	2	2	2	16	40.0
Abimbola S (2014)	2	2	3	3	3	2	4	4	23	57.5
Okpani A *et al*. , (2015)	3	3	3	3	4	2	3	3	24	60.0
Amedari MI (2021)	2	2	2	2	2	2	3	3	18	45.0
Welcome MO (2011)	2	2	2	2	2	2	2	2	16	40.0
Aregbeshola BS (2021)	2	2	2	2	2	1	2	2	15	37.5
Onwujekwe O (2019)	2	2	2	3	3	3	3	3	21	52.5
Odutolu O (2016)	2	2	2	2	3	2	3	3	19	47.5
Scott-Emuakpor A (2010)	2	2	3	3	3	2	3	3	21	52.5
Abubakar A (2022)	3	3	3	3	3	1	3	3	22	55.0
Vrushi *et al*. (2021)	2	2	2	2	2	2	2	2	16	40.0
Usman M., et al., (2022)	2	2	5	3	3	3	3	3	24	60.0
Onwujekwe O *et al*. (2020)	1	1	2	2	2	1	2	2	13	32.5
Dokunmu TM (2018)	2	2	2	2	2	1	2	2	15	37.5
PharmaAccess group, (2016)	2	2	3	2	2	1	3	3	18	45.0
Asakitikpi AE (2019)	4	3	3	3	4	2	3	3	25	62.5
Hafez R (2018)	2	2	2	3	3	3	4	4	23	57.5
Iheanacho EN (2014)	3	3	3	3	3	2	4	4	25	62.5
Abubakar M (2018)	3	2	3	3	3	2	4	4	24	60.0
Aregbeshola B (2019)	3	3	3	3	3	1	4	4	24	60.0
Hoffman LK (2017)	4	3	3	3	4	2	3	3	25	62.5
Osimen G.U., (2013)	2	2	2	3	3	3	3	3	21	52.5
Ukase P (2015)	2	2	2	3	3	2	4	4	22	55.0
Oche M (2018)	3	3	3	3	3	3	4	4	26	65.0
Kuo E (2021)	3	3	3	3	3	2	4	4	25	62.5
Transparency international (2021)	2	2	3	2	2	1	3	3	18	18.0
ADF, (2002)	2	2	2	4	3	3	3	3	22	55.0
Asuzu MC (2004)	3	3	3	3	3	2	4	4	25	62.5
Kombe G (2009)	2	2	2	2	3	2	3	3	19	47.5
Okunola A (2020)	2	2	2	2	2	2	3	3	18	45.0
Obinna O, (2020)	3	3	3	3	3	1	3	3	22	30.25
Tormusa DO (2016)	4	3	3	3	4	2	3	3	25	62.5
Oluwadare C (2013)	3	3	3	3	3	1	3	3	22	55.0
Aregbeshola BS (2016)	3	3	3	3	3	3	4	4	26	65.0
Isah EC (2008)	3	3	3	3	3	2	2	2	21	52.5
Garuba HA (2009)	3	3	4	4	4	1	4	4	27	67.5
Oyibocha EO (2014)	3	3	3	3	3	2	3	3	23	57.5
Adenuga A *et al*.2021	3	3	2	3	3	2	4	4	24	60.0
Sahara reporters, (2021)	2	2	3	2	2	1	3	3	18	45.0

**Table 3:** quality assessment of high and acceptable quality of studies

Author/year	Total score (/40)	% (High quality)
Akokuwebe ME *et al*. (2017)	30	75.0
Adegboyega K *et al*. (2012)	28	70.0
Uzochukwu BS (2015)	27	67.5
Akunne MO (2019)	24	60.0
Adebisi YA (2020)	27	67.5
Usman F (2022)	25	62.5
Anyanwu, M.U., (2020)	26	65.0
Akinwotu E (2020)	25	62.5
Amos *et al*. (2020)	24	60.0
Okpani A *et al* (2015)	24	60.0
Usman M *et al*. (2022)	24	60.0
Asakitikpi AE (2019)	25	62.5
Iheanacho EN, (2014)	25	62.5
Abubakar M (2018)	24	60.0
Aregbeshola B (2019)	24	60.0
Hoffman LK (2017)	25	62.5
Oche M (2018)	26	65.0
Kuo E (2021)	25	62.5
Tormusa DO (2016)	25	62.5
Aregbeshola BS (2016)	26	65.0
Garuba HA (2009)	27	67.5
Adenuga A *et al* 2021	24	60.0
Asuzu MC (2004)	25	62.5
Koce F *et al*. (2019)	20	50.0
Universal health coverage (2020)	16	40.0
Nigeria Centre for Disease Control (2020)	23	57.5
Okoli H *et al*. (2017)	19	47.5
Chukwuma A (2019)	20	50.0
Aregbeshola BS (2018)	16	40.0
Awosusi A (2015)	21	52.5
Olufemi J (2020)	16	40.0
Aregbeshola BS (2022)	16	40.0
Ogunkola IO (2021)	21	52.5
Obokoh A (2020)	16	40.0
Joel-Osoba G (2021)	16	40.0
This day, (2021)	16	40.0
Abimbola S (2014)	23	57.5
Amedari MI (2021)	18	45.0
Welcome MO (2011)	16	40.0
Aregbeshola BS (2021)	15	37.5
Onwujekwe O (2019)	21	52.5
Odutolu O (2016)	19	47.5
Scott-Emuakpor A (2010)	21	52.5
Abubakar A (2022)	22	55.0
Vrushi *et al*. (2021)	16	40.0
Onwujekwe O *et al*. (2020)	13	32.5
Dokunmu TM (2018)	15	37.5
Pharmaaccess group (2016)	18	45.0
Hafez R (2018)	23	57.5
Osimen GU (2013)	21	52.5
Ukase P (2015)	22	55.0
Transparency International (2021)	18	18.0
ADF (2002)	22	55.0
Kombe G (2009)	19	47.5
Okunola A (2020)	18	45.0
Obinna O (2020)	22	30.25
Oluwadare C (2013)	22	55.0
Isah EC (2008)	21	52.5
Oyibocha EO (2014)	23	57.5
Sahara reporters (2021)	18	45.0

**Main findings:** findings from the sixty articles demonstrated a wide range of in-depth narrative reviews of literature on fraud within the Nigerian health system and its response to COVID-19 pandemic. From a detailed description on the thematic content and systematic analysis, the findings were itemized in four aspects: fraud reports; assessment of fraud; the impact of fraud on health-system resilience and response to COVID-19 pandemic; and implications of fraud on healthcare service delivery amidst the ongoing COVID-19 pandemic in Nigeria. The review findings were represented further using the Agency theoretical model as follows:

**Fraud reports on response to COVID-19 pandemic in Nigeria:** the outbreak of the COVID-19 pandemic across the globe and the consequent response continues to generate heated concerns. Many apprehensions from the Nigerian health work force and international bodies are worrisome as they predicted inadequate capacity to combat the virus and its emerging variants. Another major factor that has contributed to the weakened health system is low budgetary allocation that is not adequate to purchase modern equipment and facilities [44,56,65,77,82,89,91,98]. In response to the COVID-19 pandemic in Nigeria, issues of exploitation and commercialization of COVID-19 pandemic response were determined. This was carried out through falsification of data amidst low numbers of persons tested, replication of COVID-19 healthcare facilities, sub-contracting of COVID-19 test centres, shortage of test kits/drugs [44,48,63,64,75], COVID-19 test racketeering, conflicting government reports/positions, and the apparent non-activity of COVID-19 taskforce in some part of Northern communities affected by terrorist attacks [71,84,88,93-95]. Other forms of practices of fraud within the health system are absenteeism, procurement-related fraud, under-the-counter payments, health financing-related fraud, and employment-related fraud [43,64,74,77,81,90,92,99]. In addition, regional differences played another major role in the structural and facility-level plagued with a weakened health system and destroyed by fraud and accountability issues [14,24,45,56,60,75,79]. These issues further compromised the efforts of the health workforce in carrying out their jobs as healthcare providers, including curtailing the spread of the disease. Other reports mentioned that there are high levels of distrust in the government´s role towards the poor welfare conditions for health workers and service users, and lack of proper provision of equipment for COVID-19 testing kits [62,63,86]. Thus, the fourth wave of the pandemic in Nigeria calls for sincerity of the government, health officials and citizens by fostering accountability that will surpass fraudulent practices. Thus, understanding and eradicating all forms of fraud and corruption in the health system and affecting the health system resilience requires a range of approaches that will confront this menace openly by bringing it into limelight for the glare of publicity. This will send an early warning to those who are involved or found wanting in acts of fraud to desist from them.

**Assessment of fraud in healthcare service delivery amidst the COVID-19 pandemic:** fraud in the pandemic inevitably undermines the response and deprives people of healthcare, which should be the ultimate priority of all governments [41-47]. The systematic review has shed more light on the manifestations of fraud at the point of service delivery amidst the COVID-19 pandemic in Nigeria as follows: i) Bribery to evade COVID-19 regulations: this happens when individuals made their way around social distancing regulations to evade quarantine rules by bribing officials. For instance, returnees travelling back to Nigeria from overseas were required to stay at hotels designated for total isolation at their own expense, instead of at free facilities [55-60]. As such, fraudulent practices were justified by these perpetrators at this point, as such free facilities were mismanaged, and bearing the costs was perceived as challenging and unfair [48-54]. This type of bribery ultimately leads to a further spread of the virus, aggravating the current health crisis; ii) stealing and misappropriation: Instances of stealing and misappropriation of money, medicines and other medical equipment and supplies by frontline COVID-19 healthcare teams are prevalent globally. The theft and resale of publicly-funded medicines, vaccines, and medical supplies contribute to shortages and stock-outs, limiting public health scrutiny and control, and constraining patients´ right to access adequate medical treatment. This eventually leads to the further spread of coronavirus infections and poor health outcomes, such as disability and death [73,75,87]. The pandemic has uncovered cracks in the health system, as the prevailing urgency has led many governments to relax rules, leading to lapses in sanction mechanisms [60-69]. Likewise, the distress and life-threatening fatigue health professionals have been subjected to, the low salaries and at times lack of salaries for those at the frontiers of the pandemic response, have made it a seamless background for fraudulent intent, stealing and misappropriation; iii) falsification or fabrication of data: Fraud expedited through manipulation of data is predominant in healthcare facilities. Manipulating records on services delivered, prices paid, or of health outcomes achieved, may be done to seek financial gain. Falsification of medical records includes forged billing for goods and services not provided; the creation of ´ghost patients´ to claim additional payments; and seeking reimbursements for expensive treatments that were not actually delivered [41-54]. Evidence of such practices in the context of COVID-19 is gradually emerging, as this form of fraud can affect patients directly when their needs are de-prioritized, as resources are sub-optimally allocated among different hospitals based on falsified or fabricated data. It can also influence them ultimately as remunerators. Since providers control more information, it is easy for them to manipulate data that constitutes the basis for reimbursement [52-59]. In addition, patients who submit bills to insurers directly can also engage in fraud or may connive with care providers.

**Impact of fraud on health-system resilience and response to the COVID-19 pandemic in Nigeria:** as the pandemic has uncovered major inadequacies in healthcare systems around the world, a prolonged dearth of resources combined with long, multifaceted supply chains have created unavailability of necessary medical supplies, and equipment overstretching previously stressed health systems. As cited by several studies from the 2021 independent panel on pandemic preparedness and response, several governments and health systems were inefficiently prepared for the COVID-19 pandemic, mostly in African nations [68-79]. This mirrored a legacy of abridged investment in resilient economies that left many health systems weakened and with fewer resources to cope with the sudden surge in the demand for service delivery. While many health systems found ways to respond timeously and sustain performance of the key functions of the health system, still those with resilient preliminary capacities are likely to find it easier to manage the pandemic [74-88]. The effects of fraud can be overwhelming and have the possibility of endangering the health and livelihoods of individuals and communities, including their basic needs and medical supplies. The pandemic has served as a catalyst for fraud, which has also contributed to draining the health systems [96,97]. In Nigeria, many of the reported COVID-19 related-fraudulent cases on healthcare systems were swept under the carpet, and previous fraudulent activities in the healthcare systems limited the capacity of the Nigerian health workers to respond to the outbreak at the expected time [80-85]. The pandemic outbreak has tested the health-system resilience, and for Nigeria with unequipped health systems, COVID-19 has brought her health system to the brink of failure [54-60]. In addition, it is not only that the Nigerian health system struggled to curtail the pandemic´s infection rates, but the high incidence of mortality cases from COVID-19 virus and its sub-variants majorly disrupted other key healthcare deliveries.

**Implications of fraud on healthcare delivery amidst the ongoing COVID-19 pandemic:** the emergence of theCOVID-19 pandemic has reminded us that global health should be every nation´s main concern, yet fraud remains to undermine the health systems, silently and decidedly. Since the pandemic has dispossessed millions of individuals globally of their right to access health, populations at risk are mostly affected [46-54]. The right to health comes with a series of critical commitments for governments in guaranteeing that healthcare supplies, medications, services, and facilities are obtainable, easily reached, adequate and of good quality for all, with regard to the beliefs and culture of individuals, minority interest groups, societies and communities, responsiveness to gender issues, and required progression of societal changes [52-60]. Yet, if fraud permeates service delivery, then there is little hope that government and health stakeholders can fulfil such obligations, as the unpleasant present situation with the COVID-19 pandemic is demonstrating that already. In Nigeria, failing to take cognizance of gender-related fraud in the health system among healthcare professionals in addressing fraud risks can make gender inequality and imbalances much worse, given the present pandemic situation and decaying healthcare system.

## Discussion

The manifestations of fraud at the point of service delivery in Nigeria could be debated, as this has cause decay in the Nigerian healthcare system. A bribe occurs at the point of service delivery, and informal payments are manifesting in the COVID-19 pandemic, as health systems are facing greater patient burden [8,9,12,14,45]. Thus, a dearth of reporting to date of such fraud practices during the pandemic can be due to fear of government reprisals, as those who have reported bribery activities actually faced retribution [20-24]. Several studies have been documented and reported that individuals who have experienced fraudulent activities carried out in the health sector during the pandemic do not speak out to avoid being ostracized and being denied health services in the future [10-18]. But fraud occurs in form of stealing of medicines and medical supplies such as COVID-19 diagnostic tests and personal protective items, including goggles, gloves, hand sanitizer and facemasks reported stolen by senior health authorities from COVID-19 response centers [54-85]. Several studies have given ample reports on the theft of medicines and medical supplies [41-67]. Other reports on stealing of medicines and medical supplies from hospitals are documented in studies conducted in high- and middle-income countries such as in Nigeria [67-87]. In similar manner, stealing and misappropriation occurs outside health facilities where administrative officers in charge of medical supplies facilities were allegedly involved in stealing medicines or diverting COVID-19 medical supplies, particularly during the lockdowns [71-93]. Nevertheless, absenteeism is a fraudulent practice, which still happens in the health sector, hence it should be taken seriously, and accurate data is needed to ascertain the extent of absenteeism in the health sector. At present, health workers are already in critically short supply in many countries, and health systems without sufficient human resources are unfit to deliver high quality healthcare for all [41-60]. In Nigeria´s case, absenteeism within healthcare facilities continues to happen, either as fraudulent practices or because of other forms of fraud in service delivery [22,44,52,73,87,96,97]. In addition, several Nigerian studies have reported absenteeism as a result of other forms of fraud, as several alarms have been raised in the health sector owing to the rapid spread of the virus, price gouging, hoarding, and diversion of medical supplies [65-92].

Considering the current pandemic, health workers may be under social pressure from their close-knit communities to favour them by ignoring official rules and guidelines [74-78]. If not properly addressed, these social norms will persist in vindicating such behaviors, making it difficult to improve effective measures to ensure non-discriminatory rights to use health services. The amassed influence of corrupt deeds can expend health budgets without health gains, limiting health systems´ resources intended to improve the delivery of quality healthcare for all [1-12]. With the ongoing pandemic, many developed and developing countries, including Nigeria, are seeing other forms of data fabrication and falsification, as governments and health officials are unwilling to release the factual statistics of COVID-19 cases and death rates. From the Agency theory applied in this study, what then are the probable plans to address fraud in the health sector? Our starting basis is that additional legislation is not required, as there are control institutions in Nigeria with extensive powers, as well as a raft of laws, and formal and meticulous rules and procedures, often recommended as a strategy to control the behavior of healthcare stakeholders and staff [25-34]. For instance, the 2014 National health bill and the National strategic Health development plan (2010-2015) have the Universal health coverage (UHC) constitutional mandate to strengthen the Nigerian healthcare sector by enabling oversight, accountability and governance across the three tiers of healthcare delivery through liability auditing [49-89]. Similarly, the Public Service and Public Finance Management Acts contain detailed prescripts that regulate the conduct of both public/private sector employees and the management of public/private resources. Strong laws are contingent on the political will to run fraud-free health services and delivery [1-20]. Furthermore, respect for the rule of law and an effective government machinery to enforce laws are prerequisites in the fight against fraud [41-79]. Global and national public health experts in high- and middle-income countries must engage in healthcare strategies that are open and more informed, in building effective comprehensive interventions that are sustainable, that will curb fraudulent practices in the health systems that are seriously plaguing the COVID-19 crisis, such as in Nigeria.

**Contribution to the field:** health is a fundamental human right and key indicator of sustainable development of any nation. The review highlighted the urgent need to secure the health of all citizens in order to achieve the Universal Health Coverage and Sustainable Development Goal 3 commitments in ensuring healthy lives and promoting well-being at all ages. Therefore, this study contributed by identifying the peculiarities in fraud from previous studies in ascertaining the implications of not taking a bold commitment to end fraud amidst the burden of diseases, including COVID-19, in Nigeria via a systematic review approach.

**Study limitations:** this study has several strengths. Firstly, the sample size of the articles included for the study was large enough to conduct a robust systematic review analysis and produce thematic content findings. Secondly, the study provided a representative sample of sixty articles to produce generalizable results with regard to fraud within the health system amidst the COVID-19 pandemic in Nigeria and proffer sustainable solutions to address fraud-related activities. This is crucial for sustaining resilience in the health system, by introducing specific effective preventive interventions to curb fraud in the healthcare system. However, the study is not without its limitations. The thematic content analysis of the articles sampled on fraud may have been biased, as some of the outcomes from these articles may tend to omit, under-report, or over-report results.

**Recommendation:** based on the data and findings, the authors provide the following recommendations: first, incorporating anti-corruption platforms (such as transparency, accountability, integrity) will create an involvement in wider efforts to strengthen assessments and national health planning exercises in addressing all forms of fraud rooted in COVID-19-related programmes, plans and policies in Nigeria. Implementing proper sanctions and enforcement of transparency and accountability will defend prioritized processes in allotting COVID-19 vaccines, palliatives, and distribution of relief materials. Second, monitoring data and other COVID-19-related information is important to address all forms of fraud. Non-governmental organizations in Nigeria need to assist in sensitizing the public on fraudulent activities in the health information systems by collecting and sharing the data, as this will aid in improving countries’ emergency response readiness for the ongoing COVID-19 pandemic and other future health emergencies. Similarly, documentation of fraud cases found at the point of delivery of health services will serve as pressing evidence to advocate for transparency and accountability platforms that will guarantee a more equitable access to healthcare services.

## Conclusion

Fraud in the Nigerian health system is associated with weak governance, and the COVID-19 pandemic ought to have marked a critical point in time in health systems to identify and ameliorate the health system resilience´s vulnerabilities to fraud. The Nigerian government and relevant health stakeholders lack political will and commitment in addressing the pathetic situation. However, specific fraud interventions should be geared towards addressing fraud issues which exploit the vulnerable and weakened health system, service delivery and high-risk institutionalized health workforce within the Nigerian health system.

### What is known about this topic

- *Existing studies in Nigeria have documented that fraud within the healthcare system has been a long-existing but ignored pandemic threating the universal health coverage (UHC) of the Sustainable Development Goal (SDG) 3;*
- *The vulnerability of the Nigerian health system had not been given much attention to prior to the pandemic, which has raised serious concerns, and how to respond to the pandemic with a weak health system has not been well documented in Nigeria*.

### What this study adds

- *This study showed that the Nigerian government should act on the lessons learnt from the devastating COVID-19 pandemic; Government renewing its commitments to health investment and building strong health systems for resilience in the face of any future health threats must be taken into consideration in addressing fraudulent activities operating within the Nigerian health system*.
